# Spatial Acuity and Prey Detection in Weakly Electric Fish

**DOI:** 10.1371/journal.pcbi.0030038

**Published:** 2007-03-02

**Authors:** David Babineau, John E Lewis, André Longtin

**Affiliations:** 1 Department of Physics, University of Ottawa, Ottawa, Ontario, Canada; 2 Department of Biology, University of Ottawa, Ottawa, Ontario, Canada; 3 Center for Neural Dynamics, University of Ottawa, Ottawa, Ontario, Canada; University College London, United Kingdom

## Abstract

It is well-known that weakly electric fish can exhibit extreme temporal acuity at the behavioral level, discriminating time intervals in the submicrosecond range. However, relatively little is known about the spatial acuity of the electrosense. Here we use a recently developed model of the electric field generated by Apteronotus leptorhynchus to study spatial acuity and small signal extraction. We show that the quality of sensory information available on the lateral body surface is highest for objects close to the fish's midbody, suggesting that spatial acuity should be highest at this location. Overall, however, this information is relatively blurry and the electrosense exhibits relatively poor acuity. Despite this apparent limitation, weakly electric fish are able to extract the minute signals generated by small prey, even in the presence of large background signals. In fact, we show that the fish's poor spatial acuity may actually enhance prey detection under some conditions. This occurs because the electric image produced by a spatially dense background is relatively “blurred” or spatially uniform. Hence, the small spatially localized prey signal “pops out” when fish motion is simulated. This shows explicitly how the back-and-forth swimming, characteristic of these fish, can be used to generate motion cues that, as in other animals, assist in the extraction of sensory information when signal-to-noise ratios are low. Our study also reveals the importance of the structure of complex electrosensory backgrounds. Whereas large-object spacing is favorable for discriminating the individual elements of a scene, small spacing can increase the fish's ability to resolve a single target object against this background.

## Author Summary

Extracting and characterizing small signals in a noisy background is a universal problem in sensory processing. In human audition, this is referred to as the cocktail party problem. Weakly electric knifefish face a similar difficulty. Objects in their environment produce distortions in a self-generated electric field that are used for navigation and prey capture in the dark. While we know prey signals are small (microvolt range), and other environmental signals can be many times larger, we know very little about prey detection in a natural electrosensory landscape. To better understand this problem, we present an analysis of small object discrimination and detection using a recently developed model of the fish's electric field. We show that the electric sense is extremely blurry: two prey must be about five diameters apart to produce distinct signals. But this blurriness can be an asset when prey must be detected in a background of large distracters. We show that the commonly observed “knife-like” scanning behaviour of these fish causes a prey signal to “pop-out” from the blurry background signal. Our study is the first to our knowledge to describe specific motion-generated electrosensory cues, and it provides a novel example of how self-motion can be used to enhance sensory processing.

## Introduction

Weakly electric fish are commonly found in the freshwater systems of South America and Africa [1,2]. These nocturnal fish use a unique sensory modality, called the “electrosense,” to help them navigate, communicate, and find prey in the absence of strong visual cues [3]. The electrosense involves a specialized electric organ that emits an electric discharge resulting in a dipole-like electric field in the surrounding water [4]. The transdermal potential (the so-called “electric image”) is continuously monitored via electroreceptors found in the skin layer. Changes in the spatial properties of the electric image can provide cues that help the fish determine the location, size, and electrical properties of nearby objects [5–10].

Recent studies have shed new light on the weakly electric fish's perceptual world. In the context of distance perception, the amplitude and width of an electric image were shown to be analogous to visual contrast and blur [11]. The electric image produced by an object can also be distorted by nearby objects; consequently, conductive objects can act as electrosensory “mirrors” [12]. In contrast with the visual sense, however, the electrosense has no focusing mechanism and is limited to the near-field, so it is generally considered a “rough” sensory modality [13–16]. In fact, the range of active electrolocation in weakly electric fish is likely only about one body length [7], and considerably less for small prey-like objects [17]. Within this range, much is known about the fish's temporal acuity [18,19], but relatively little is known about the fish's ability to resolve multiple nearby objects.

Here, we consider the notion of “electro-acuity,” analogous to the notion of visual acuity found in the visuo–sensory lexicon, to investigate the quality of electrosensory information in the spatial domain. A common measure of acuity in other sensory systems is the just-noticeable difference, or the minimum difference between two stimuli such that they are perceptually distinct [20]. In the present context, we consider an analogous measure to describe the quality of electrosensory input available for a discrimination task. We define this measure as the minimum spatial separation of two objects (S_min_), such that two distinct peaks remain in the electric image on the fish's skin (Figure 1). Using a 2-D finite element method model of A. leptorhynchus' electric field [9], we show that S_min_ is smallest in the fish's midbody and decreases for objects placed farther away from the fish. This suggests an interesting contrast with the “electrosensory fovea” in the head region [10,17], where the highest density of electroreceptors is found [21]. Overall, we found that electroacuity is poor relative to visual acuity in humans, but is comparable with that of the human somatosensory system.

**Figure 1:**
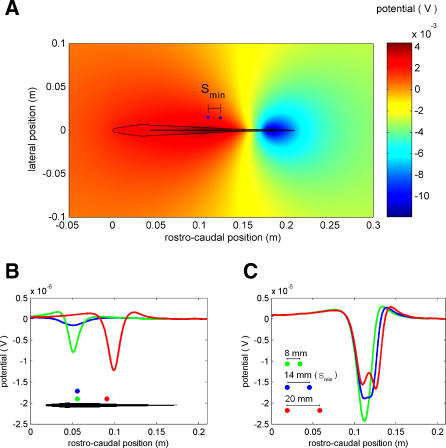
Electric Images Produced by Two Prey-Like Objects and Determination of *S_min_* The head is at position 0 m along the rostro–caudal axis. The midbody is at 0.11 m, and the tail is at 0.21 m. All interobject distances are center-to-center, and object-to-fish lateral distances (i.e., perpendicular to fish midline) are from object center to skin surface. (A) Electric field potential in the presence of two identical prey-like objects (modeled as 0.3-cm diameter discs with a conductivity of 0.0303 S/m; water conductivity: 0.023 S/m). Objects do not affect the field much due to their small size and conductivity similar to the water. The *S_min_* (14 mm) is also shown for a specific prey position (left prey located 0.11 m caudally from the tip of the head and 0.012 m laterally to the skin). The potential at different points is measured with respect to a reference electrode placed laterally to the fish in the far field, near the zero potential line [9]. (B) Overlays of electric images for three different object locations illustrating the increase in image amplitude in the caudal direction (*x*) and the decrease in amplitude for increasing lateral distances (*y*). (*x*,*y*) = (0.05, 0.03), (0.05, 0.015), (0.1, 0.015) m. As described in Materials and Methods, these images are computed as the difference between the transdermal potentials measured with and without the object present. (C) Overlays of electric images for three distinct interprey distances (see inset). Blue trace shows *S_min_*, when the two peaks in the electric image are just noticeable. Computation of the images is as in (B). Location of more-rostral prey as in (A).

Despite the apparent low quality of electrosensory signals, weakly electric fish are able to detect small prey [7,17]. Although there is no direct evidence, it is reasonable to assume that they do so even in the presence of noisy background signals [7]. In a related task (object tracking), background noise has been shown to degrade performance [22,23]. Single-cell recordings in midbrain neurons have further revealed that some low-frequency background signals can interfere with directional selectivity [24]. It is thus believed that some of the natural behaviors exhibited by the fish play a central role in signal extraction. In particular, simulations have suggested that tail-bending could improve object detection by increasing the electric image's amplitude [13,14].

It has also been suggested that the back-and-forth swimming, or scanning motion, observed in these fish could be used to generate specific electrolocation cues [25–28], although this has not yet been demonstrated. Indeed, to elucidate the nature of these motion-related cues, we have simulated this scanning motion and show that, under some conditions, this behavior could assist in extracting small prey-like signals from large background ones. We show that the component of the electric image produced by a sufficiently dense background does not change during scanning, whereas the one produced by the prey object, albeit miniscule in comparison, does. This process is similar to motion-related cues and active sensing techniques seen in other contexts [28,29].

## Results

In the following analyses, we use our previously described finite-element model of the electric field produced by A. leptorhynchus (see Materials and Methods and [9,30])*.* Figure 1A shows the simulated dipole-like potential map for this fish in the presence of two prey-like objects. Such objects do not greatly perturb the fish's natural field due to their small size and conductivity (which is similar to that of the water). Figure 1B shows overlays of electric images due to single objects at different locations (i.e., each image is computed separately). Such images show characteristic shapes but vary systematically in amplitude and width with rostral–caudal and lateral location [5,9,10]. Figure 1C shows images produced by object pairs for three different interobject distances (shown in inset). Prey-like objects that are located too close together (green trace) produce a single peak in the electric image (similar to the images in Figure 1B), while objects separated by a larger distance produce two distinct peaks (red trace). The blue trace illustrates the electric image in which two peaks are just barely distinguishable; we define the associated interobject distance as S_min_. Thus, S_min_, measured in these noiseless conditions, delineates a limit to electroacuity. A smaller S_min_ suggests better electroacuity (i.e., increased spatial resolution). For this specific prey-like object and rostro–caudal location, the S_min_ is 14 mm. This suggests that, at this lateral distance, these two objects must be separated by at least 14 mm, a distance approximately five times their diameter, to be distinguished.

Electroacuity varies for different lateral and rostro–caudal object locations (Figure 2, see insets). Figure 2A and 2C shows the effects of object size and conductivity, respectively, on electroacuity for different lateral positions (rostro–caudal position fixed near the fish's midpoint, 0.11 m). S_min_ increases (electroacuity decreases) for objects that are placed farther away from the fish, regardless of object size or conductivity. When objects are far from the fish, S_min_ is roughly independent of object size (Figure 2A). At the closest location possible for the largest object (blue curve), S_min_ is smaller than for the other object sizes. This is a consequence of the relative sharpening of the image for close large objects (see Figure 1B). The sharpness of an image can be quantified by the reciprocal of its normalized width (width divided by amplitude). Image sharpness decreases (normalized width increases) with lateral distance and, in general, is independent of object size [5]. However, object size becomes a factor for locations close to the skin (see largest object in Figure 2A and 2B), as larger objects produce relatively sharper images in these cases [9]. Note also that there is a slight inflection at a lateral distance of 0.016 m (Figure 2A and 2C) due to the spatial heterogeneity of the electric field (higher density of field lines near the zero potential line, which curves rostrally as seen in Figure 1A).

**Figure 2:**
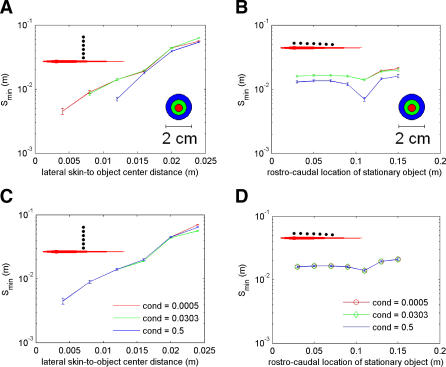
Effect of Object Location and Conductivity on Spatial Electroacuity In all panels, see fish insets for approximate lateral and rostro–caudal locations where *S_min_* was calculated. Error bars represent the sampling that was used to calculate the *S_min_* (either 0.5 or 1 mm). Lateral distance is measured as object center to fish skin (as in Figure 1). (A) Effect of lateral distance on *S_min_* for three distinct object diameters (rostro–caudal location, *x* = 0.11 m). Red, 0.3 cm (prey size); green, 1 cm; blue, 2 cm. Object conductivity fixed at 0.0303 S/m (prey conductivity). (B) Effect of rostro–caudal position on *S_min_* for same object sizes and conductivity as (A), with a lateral distance of 0.012 m. (C) Effect of lateral distance on *S_min_* for three distinct object conductivities (rostro–caudal location, *x* = 0.11m). Red, 0.0005 S/m (plant conductivity); green, 0.0303 S/m (prey conductivity); blue, 0.5 S/m. Object diameters fixed at 0.3 cm (prey size). (D) Effect of rostro–caudal position on *S_min_* for same object diameter and conductivities as in (C), with a lateral distance of 0.012 m.

Figure 2B and 2D shows the effects of object size and conductivity, respectively, on electroacuity for different rostro–caudal positions (lateral object center-to-skin distance fixed at 0.012 m). In general, S_min_ is smaller for larger objects, all along the length of the fish. The largest objects (2 cm) can actually be distinguished in the artificial condition of overlapping (i.e., the two objects are fused into a single composite peanut-shaped object), suggesting a mechanism for shape discrimination under some conditions. The position *x* = 0.11 m suggests a point of optimal acuity along the side of the fish. The two peaks in the image can be distinguished more easily for objects in this region because this is the rostro–caudal location where electric images are sharpest [9,10], so that there is minimal interaction between the individual images produced by each object. Object conductivity has comparatively little effect on the S_min_ in both lateral and rostro–caudal directions (Figure 2C and 2D). Overall, S_min_ varies much more in the lateral direction than in the rostro–caudal direction (compare Figure 2A–2C and 2B–2D) due to the relatively large changes in image sharpness as lateral object distance increases [5,8].

The effect of water conductivity on electroacuity was also studied for a specific location (*x* = 0.11 m, *y* = 0.015 m). For the range of water conductivity values found in the rivers in which A. leptorhynchus live (between 0.00085 and 0.01135 S/m [2]), S_min_ changes only slightly. As an overall trend, S_min_ decreased as water conductivity diminished (from 15.5 to 12.5 mm as water conductivity decreased from 0.05 to 0.0005 S/m).

As a first step toward understanding electroacuity in a more natural context, the electric images produced by differently sized arrays of background objects (with “plant-like” conductivity) were studied systematically. In Figure 3A, the red trace shows the electric image produced by a single such object located 0.11 m caudally from the tip of the fish's head (red object in inset located close to the fish's midpoint). The orange trace shows the electric image produced by three objects: the central one (red) plus one (orange) added 0.03 m on each side. In a similar progression, electric images are shown for up to 11 objects. With larger numbers of aligned objects, the electric images converge. Thus, for an array of seven objects (approximately a fish body length), the image is almost the same as with 11 objects. The electric images are each marked by a singular peak because the interobject distance is too small (at this lateral distance of 0.05 m) to resolve different peaks, i.e., object separation is less than S_min_. The small bumps at approximately 0.03 m and 0.2 m are due to abrupt changes in fish geometry near the head and tail, respectively, and are not due to individual objects within the background array. Similar results were also observed for object arrays positioned closer to the fish, where different peaks were observed in the electric image, as well as for solid bars of increasing widths (unpublished data). Figure 3B shows the effect of changing the object spacing in similar arrays. At the largest spacing (red), the image is dominated by the contribution from the central object. For arrays that are more spatially dense (green, blue), the contributions of individual objects are blurred, resulting in an image with a broad peak.

**Figure 3:**
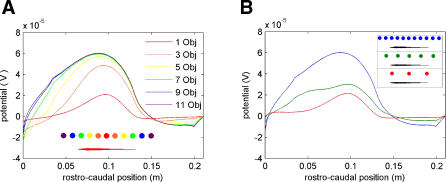
Electric Image of a Plant-Like Background All images in both panels are computed as the difference in transdermal potentials, with and without objects (as described in Materials and Methods). (A) Electric images produced by six distinct background widths, which differ in number of objects (see inset). Red, 1; orange, 3; yellow, 5; green, 7, blue, 9; purple, 11. The 2 cm–diameter discs have a conductivity of 0.0005 S/m to mimic the plant Hygrophilia. Discs are located 0.05 m away laterally from the fish's midline and are separated, one from another, by 0.03 m. All series of objects are centered near the fish's midpoint (red object in inset) and color in inset denotes external objects of a given series. (B) Electric images due to backgrounds with three different interobject spacings: blue, 0.03 m (same as panel A); green, 0.06 m; red, 0.09 m. Otherwise, objects are identical to those in panel (A).

These object arrays provide a simplified model of the background signals comprising a natural electrosensory landscape. To better understand how weakly electric fish are able to detect miniscule prey in the presence of large-background signals, we calculated the electric image produced by a small Daphnia-like prey object against a large-background array of objects (Figure 4). Even though the prey is located just 0.008 m from the fish's skin (compared with the 0.05 m lateral position of the background), the electric image with the prey and background is not much different than the one obtained with the background alone (largest deviation between the two images is about 4%; compare Figure 4A and 4B). The interesting feature, however, is that the overall image shape is similar regardless of the fish's position during a simulated scanning movement (even though the background was simulated as a discrete set of objects). This can be understood in terms of electroacuity: the background objects are too close together to be distinguished and thus form a blurred image. It is critical to note that during the scan, however, the small blip created by the prey *does* change location within the electric image (Figure 4B; note that the images do not overlap perfectly). Next, we demonstrate this point explicitly by considering the time-varying image during a simulated scanning movement.

**Figure 4:**
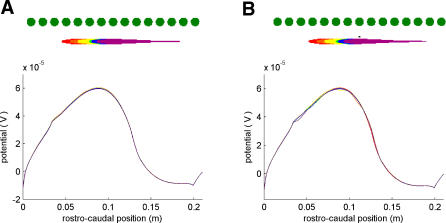
Electric Image of a Plant-Like Background in the Presence and Absence of a Prey Object All images in both panels are computed as the difference in transdermal potentials, with and without objects (as described in Materials and Methods). (A) Six fish positions (see inset, top) for which the electric images (bottom) produced by a 15-disc Hygrophilia plant-like background (0.05 m lateral to fish, as in Figure 3) were calculated. Electric images are barely distinguishable from one another. Fish positions differ from one another by 0.02 m, 0.02 m, 0.03 m, 0.005 m, and 0.015 m (see inset). (B) Same as in panel (A) except a Daphnia-like prey object (0.3-cm diameter as in Figure 1) was added at a lateral distance of 0.008 m from the skin.

The consequence of the relative differences between background and prey during a scanning movement is that the small prey signals can be extracted by looking at the time-varying transdermal potential at specific locations along the fish's body. Figure 5 illustrates the temporal profile of the transdermal potential at two distinct body locations under different conditions. The signal measured at Location A (see inset) reveals a clear prey-dependent component (Figure 5A, compare green and blue traces). Note also that this prey signal (in the presence of the background) is very similar to that for the prey-alone condition (Figure 5A, compare blue and red traces). When the interobject distance in the background becomes too large, as in Figure 5B, the background image is no longer blurred and individual object characteristics appear, thereby masking the prey-specific signal. This effect can be even more pronounced when the objects are randomly spaced over the same area (Figure 5C). Figure 5D–5F shows a similar result for a different body location (note that the prey-specific signal occurs slightly later in time at this location, due to the scanning direction).

**Figure 5:**
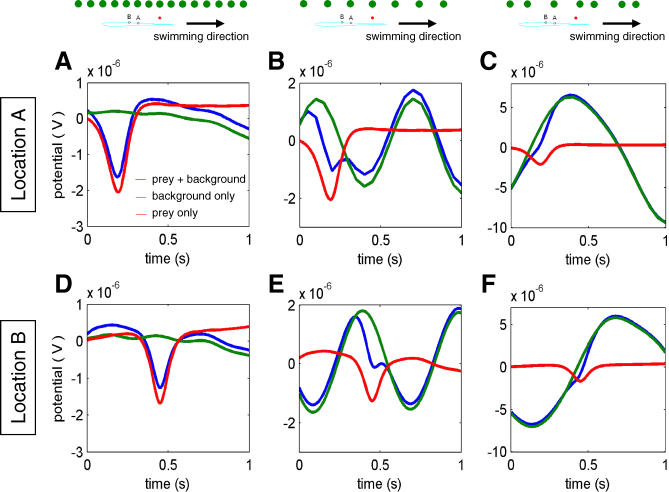
Transdermal Potential at Two Distinct Points on the Fish's Body During Simulated Motion To calculate each image, 21 different fish positions were used. In all cases, images are the raw transdermal potential with the mean removed to more easily compare the different curves. Black arrow shows direction of the simulated scanning motion used to generate the time series shown, with a scanning speed of 0.1 m/s. The legend in (A) applies to all panels. (A) Transdermal potential at a skin location 0.11 m caudal from the tip of the fish's head (point A in inset) for three different conditions: background alone (green), the background and prey (blue), and prey alone (red). Background objects are as in Figures 3 and 4. The spacing between the individual objects in the background is 0.03 m; the lateral distance of the background is 0.05 m from the midline. The lateral distance of the prey object (as in Figure 4) is 0.008 m. (B) Same as in panel (A) except for a larger interobject spacing (0.06 m) in the background. (C) Same as in panel (A) except that the background objects are randomly spaced, as shown by the inset, with same mean spacing as (B). (D–F) Same as the upper panels (A–C, respectively) except that the transdermal potential is shown for a skin position 0.085 m caudal from the tip of the fish's head (point B in inset).

Figure 5A and 5B suggests that as the objects within the background are increasingly separated, the prey will be less distinguishable. We confirm these observations in terms of a signal-to-noise ratio (SNR) of prey signal versus background (see Materials and Methods). The SNR decreases with increasing interobject separation in the background (Figure 6; left axis, blue trace). For reference, we can compare this situation with the expected discriminability of two *individual* objects (see Materials and Methods), where the electric image components due to each object become increasingly distinct as the objects are moved apart (Figure 1B; Figure 5C: right axis, green trace). This applies to the case of two prey-like objects in the absence of background, as in Figure 1A and 1C and Figure 2, as well as to the case of two background-like objects. In a more natural context, the blurriness of the electrosense interestingly has the effect of enhancing sensory performance. And indeed, this should apply to a wide range of electrosensory landscapes, as blurriness will be unaffected by small changes in object conductivity (Figure 2C and 2D).

**Figure 6:**
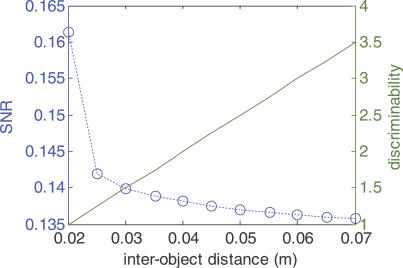
Prey Detectibility and Background Sparseness (Left axis, blue trace) SNR ratio between the prey and background transdermal potential time series and the background-only time series (i.e., between blue and green traces in Figure 5A; see Materials and Methods for more details). Each point represents the mean SNR of ten locations (over an ~0.01 m–wide patch of skin) centered 0.05 m caudal from the tip of the fish's head. SNR is shown as a function of interobject spacing of the background. (Right axis, green trace) Theoretical discriminability (see Materials and Methods) between two background-type objects as a function of their spacing, using the same object size (2-cm diameter) and lateral distance (0.05 m) as in Figure 5.

## Discussion

The extraction of small environmental signals is a problem faced by all sensory systems. The mechanisms by which this problem is solved have been studied extensively, not only in the human senses, but also in sensory modalities unique to other species [28,31]. Indeed, the electrosensory system exhibits many parallels with other senses, including human vision and audition [11,32], but we know relatively little about small-signal extraction and the spatial resolution of this modality. Here, we have considered these aspects of electrosensory processing in terms of the primary sensory input as a first step toward understanding acuity and object detection at the behavioral level.

### Electroacuity Measurement

Many recent studies have contributed to our understanding of electrosensory scene analysis [9,26,27,33,34]. In particular, Rother et al. [12] have shown that the electric image due to two objects is the result of complex interactions between the effects of each object. To extend these studies in the context of object discrimination, we have introduced the notion of electroacuity. This measure, comparable to the notion of visual acuity, has helped us quantify the “sharpness” of the electrosense in the spatial domain. Studies have suggested that this was a rather “rough” sensory modality [7,14], and our findings, in terms of the sensory input, confirm this quantitatively. For example, we found that two prey-like objects located within the range of natural prey detection (which is typically less than 20 mm, [17]), must be separated by 9 mm for the electric image to show features of both objects (Figure 2). We characterize this limit by the quantity S_min_, analogous to the psychophysical notion of the just noticeable difference and the Rayleigh criterion in optics (see Materials and Methods). Electroacuity is much lower than human visual acuity [35]. In contrast, the electrosense fares much better when compared with tactile two-point discrimination in humans, where thresholds are as high as 50 mm in some body locations [36,37].

The magnitude of S_min_ will increase with the disparity in both the image amplitudes and widths for the two objects. It will also be influenced by nonlinear effects between image amplitude and image width for close pairs of objects (which our simulations implicitly capture), but we have not systematically investigated them here (but see [12]). That said, to a reasonable approximation, S_min_ is proportional to the normalized width of the image due to each of the objects (see Materials and Methods).

Figure 2B shows that for locations in the rostral half of the fish, S_min_ changes relatively little. This interesting feature is primarily due to the uniformity of the field in this range: the current lines are nearly perpendicular to the fish body axis. The field uniformity is a result of the spatial filtering effects (smoothing) due to the tapered body shape [9,10,38]. This means that the spatial extent of an object's influence on this field (image sharpness) will be relatively constant. For locations closer to the midbody, the field lines are more concentrated (i.e., the field is not as uniform as for more rostral locations), so the influence of the object is more focused. The image amplitude also increases in this range of body locations (Figure 1B; Figure 5 of [9]), further contributing to a sharper image. However, as outlined in detail in Materials and Methods, although the image amplitude increases, then decreases, in the rostro-to-caudal direction [9], S_min_ is determined by image sharpness (normalized image width) and is much less sensitive to absolute amplitude (Figure 2B, compare red and green traces).

In terms of the quality of sensory input, our results reveal a point of optimal electroacuity located in the fish's midbody. This is in contrast to the notion that optimal discrimination should occur near the fish's head region, the electrosensory fovea, which has the highest density of electroreceptors [21]. However, determining acuity in the head region is a complex task due to a number of factors. For example, some enclosed environments can interact with this geometry and produce an electric “funneling” effect that increases the local field amplitude and enhances object discrimination [39,40]. Although these studies were performed on a different species of electric fish (pulse-type discharge) with a different electric organ morphology, a detailed investigation of the head region in A. leptorhynchus (the species we consider here) is still warranted. This will, however, require a more complicated 3-D model, so determining how the electric field, body geometry, and receptor density combine to determine electroacuity in the electrosensory fovea is not possible at this time. Nevertheless, on the lateral body surface, the combination of body geometry and current density are such that electric images are sharpest in the midbody [9], thus allowing the objects to be closer in that region before their electric images blur and form a single peak. This apparent tradeoff between more receptors rostrally and higher-quality images caudally may explain why prey detection occurs at approximately equal rates over all rostro–caudal locations [17].

An additional consideration, which again points to interesting future research, is that our current model does not account for the electric field dynamics that could in principle cause midbody acuity to vary over the electric organ discharge cycle. It is possible, for example, that the lowest S_min_ seen here in the midbody region may shift to other locations for other phases of the cycle, due to the spatial variation of the field in time [38].

In a strict sense, the values we obtain for S_min_ can be considered as an upper-bound limit on spatial acuity, since various noise sources would undoubtedly result in lower acuity at the behavioural level. However, there are additional cues available from the electric image, and potentially from other sensory modalities, which could help distinguish adjacent objects, and hence increase acuity. Specifically, the electric image produced by two objects is still wider than the image of one of the objects alone, even when their individual peaks are not discernable (see Figure 1C). Moreover, we have only considered two adjacent objects located in parallel with the fish's contour. Indeed, different criteria are required to measure the discrimination of objects that are situated one-behind-the-other (i.e., perpendicular to the fish's contour). Rother et al. [12] have studied such object locations, but not in the context of spatial acuity.

We have shown that electroacuity did not vary with object conductivity. This implies that the fish's ability to resolve two equally sized, equally conductive objects is the same, regardless of whether these objects are animate or inanimate. However, it is possible that the addition of environmental noise to the electric images would make one of these types of objects more “resolvable,” as the SNR would be greater for high-conductivity objects. Water conductivity, on the other hand, does (slightly) affect S_min_. Our results are in accord with other findings, which state that object detection is best-achieved in low-conductivity water [17,41,42], confirming the notion that increased water conductivity acts as a type of electrosensory “fog.”

To resolve all of these issues, further behavioral experiments are required. Our current studies using a 2-D electric field model [9] have generated many hypotheses to test in such experiments. Despite the fact that the 2-D model very accurately reproduces many spatial aspects of the electric field [9], ultimately a more detailed 3-D model of the time-varying electric field will be necessary. Measuring electroacuity (behaviorally) in these fish could be accomplished by using a forced-choice experimental paradigm. In this task, the fish could be trained to choose between a single object and a pair of objects, with a reward given for the choice of the latter. An estimate of electroacuity could be obtained by tracking the accuracy of the choices as the interobject distance was decreased (see [33,43,44] for similar protocols).

### Prey Detection in Weakly Electric Fish

Weakly electric fish are subject to a wide range of stimuli in natural electrosensory landscapes. Large conducting boundaries, such as rocks or the river bottom, constitute extensive background clutter [27]. The fish therefore has the challenging task of extracting small prey signals from enormous background ones. To investigate this scenario, we have modeled a plant-like background. We have shown that, as this background increases in width, the electric images produced on the fish's skin converge (i.e., the images are blurred). In fact, the image is not much different for background arrays ranging from 0.18 m to 0.3 m wide. In the presence of such a large-background image, the S_min_ for prey objects would be much larger than for the conditions we have considered thus far, and may in fact be defined only for much larger objects. In other words, as discussed in the following, the electric image component due to the background obscured that due to the two small prey-like objects.

Figure 4 clearly indicates that the effect of a prey is miniscule in the presence of a relatively large-background array. Even at different times during a simulated scanning behavior, the prey only affected the image due to the background by a few percent at most. This suggests that for any static “snapshot” the fish would not be able to extract the prey signal from the large-background signal. On the other hand, weakly electric fish are known to detect minuscule signals under some laboratory conditions [17,45], and presumably can do so in the wild while hunting. We suggest that movement is required in these situations. In fact, due to the blurring effect, the background component of the electric image does not change with fish scanning, whereas the prey component does (see Figure 4B). As a consequence, the small-prey signal is revealed during the scanning motion by looking at the transdermal potential at individual locations on the fish's body (Figure 5A and 5D). In contrast, when background objects are more separated, the prey signal remains confounded by the background (Figure 5B, 5C, 5E, and 5F).

The separation of small signals from background is a universal problem in sensory processing. In vision, the so-called figure-from-ground separation has been extensively studied; luminance and contrast differences between figure and ground provide information-rich cues for this task. In the absence of such cues, however, relative motion (due to figure, background, or observer motion) can provide information that is critical for effective figure-ground separation [29,46]. Motion of an auditory stimulus can also provide cues for sound-source localization in a noisy background [47,48]. Though the particular mechanisms involved in each sense may differ [47], both rely on coherent changes in stimulus parameters (spatial correlation in vision, systematic sweep of interaural time delays in audition). Similarly, we have shown that motion can also lead to small-signal detection in an electrosensory landscape under certain conditions. When the constituent objects of a complex scene are close enough to each other to result in a blurred (spatially uniform) image, a small spatially localized prey signal will pop out due to motion cues (and without motion the prey signal is masked by the large background). On the other hand, to evaluate the specific features of a scene, a greater spacing among constituent objects is required (see Figure 6).

### Electrosensory Processing

It is important to note that we have only considered the information available to the electrosensory system and have not considered the potential for extracting this information. Information encoded by individual electroreceptor afferents will be pooled in the hindbrain electrosensory lateral line lobe (ELL). Here, the principle neurons, ELL pyramidal neurons, have receptive fields that vary systematically in size across three somatotopic maps. The largest of these receptive fields (lateral segment map) are about 2 cm in width along the body axis of the fish; the smallest receptive fields (centromedial segment map) are about 0.5 cm in width [26,49]. As previous studies have shown, the different maps may take on different roles depending on the type of information available [26,50]. In the context of this paper, the most focused images due to nearby prey objects may be preferentially encoded using pyramidal neurons of the centromedial segment (smaller receptive fields), and the more blurred images due to background objects may be encoded by neurons of the lateral segment (larger receptive fields).

In addition, there are mechanisms in the ELL (via feedback pathways) that can cancel out predictable or redundant stimuli [51,52]. In principle, when the background is spatially uniform (blurred), such feedback mechanisms could cancel out the large-image component due to the background and further enhance small signal extraction during scanning. Recent studies on the signal processing features of ELL neurons have shown that coherence to spatially global time-varying input is high-pass [53], suggesting again that responses to spatially dense backgrounds can be filtered out. Information encoded by ELL neurons is transmitted to higher-order neurons of the midbrain. Recent studies have described plasticity and motion sensitivity in these neurons [24,54], but further studies will be required to determine how these neurons contribute to the computations involved with prey detection and discrimination in complex landscapes.

### Conclusion

It has been widely hypothesized that the stereotypical back-and-forth scanning behavior exhibited by weakly electric fish could be used to generate electrolocation cues [25,55,56]. In fact, cues obtained by self-motion are used by many different animals to extract relevant sensory features [28]. For example, primates move their fingers laterally to detect fine features in textured surfaces, which would otherwise go unnoticed [57]; rodents perform whisking behaviors [58]; and insects, such as mantids, can obtain information about an object's depth using a side-to-side “peering” movement (by means of motion parallax cues; [59]). Such examples have led to the reasonable notion that the exploratory behaviors exhibited by weakly electric fish, such as the aforementioned scanning, act similarly to provide relevant information from complex electrosensory scenes. Our study describes the nature of these motion-generated cues for the first time, and indeed shows that their effectiveness depends on context.

In particular, our results predict that weakly electric fish should exhibit the specific search behavior that is most suitable for signal extraction in a given context. The scanning behavior would be best suited for spatially dense or uniform backgrounds, whereas the fish might preferentially use tail-bending in cases where the background is sparse (as in Figure 5B, 5C, 5D, and 5F where the prey component is confounded with the background signal). In future studies, we aim to determine which behaviors are used most frequently by the fish to explore electrosensory landscapes with varying spatial characteristics.

## Materials and Methods

The 2-D electric field of a 21-cm A. leptorhynchus was simulated using a finite-element–method model described previously in [9]. Briefly, the model reproduces the field measured at one phase of the quasisinusoidal electric organ discharge. It consists of three components: an electric organ (EO), a body compartment, and a thin skin layer. The EO current density and the conductivities of the three components were optimized using raw data provided by Christopher Assad [38]. The optimized EO current density is spatially structured; as compared with a simple dipole, it is skewed toward the tail. Such a profile in the EO current density, as well as the spatial filtering due to the tapered body shape, reproduces the asymmetric “multipole” electric field [9,10,27]. To distinguish this situation from that of a simple dipole, we sometimes refer to the fish's electric field as “dipole-like.” This model is a 2-D simplification that is static in time, and so, in principle, any results derived from it are qualitative. It is important to note, however, that the model provides a quantitatively accurate representation of the data measured in the horizontal plane [9], and thus should be very reliable. Of course, as we note in the Results and Discussion sections, there are some questions that will require a detailed time-varying 3-D model.

Electric images were calculated in one of two ways using custom MATLAB subroutines. In Figures 1–4, images are defined as the differences in transdermal potential, with and without objects present (this has become the standard definition of an electric image, [5]). In Figure 5, images are displayed as the raw transdermal potential, the natural electrosensory input. All images are shown only for the side of the fish body closest to the objects. Water conductivity was set to 0.023 S/m, as in [38]. The prey chosen, Daphnia magna, was modeled as a 3 mm–diameter disc with a conductivity of 0.0303 S/m, as in [15,17]. The background objects (2-cm discs) simulated throughout this paper were based on the conductivity of the aquatic plant Hygrophilia [22] (0.0005 S/m). The goal was not to mimic the plant's geometry accurately, but rather to get a general idea of the effects caused by varying backgrounds with plant-like conductivity and size.

To estimate the fish's ability to resolve two distinct objects (electroacuity), the minimal distance S_min_ was calculated. This measure is the interobject distance, which delimits an electric image with one peak from one with two peaks (for example, see Figure 1C). This quantity depends on a number of parameters such as the object's size, its rostro–caudal and lateral location, and the water conductivity. We can develop more intuition for how S_min_ behaves assuming that images of objects are idealized Gaussians. Consider two Gaussians along the *x*-axis, of similar standard deviation σ and amplitudes, but centered on μ_1_ and (−μ_1_ ), respectively. Assuming linear superposition, their sum along the *x*-axis will have one or two maxima, depending on the relation between the standard deviation and the mean, i.e., on the relative width. It can be shown that S_min_ in this case corresponds to (2σ). If the amplitudes of the Gaussians change in the same way, as they do when the object is closer to the fish, S_min_ remains the same; it will increase, however, if there is disparity in the amplitudes. S_min_ will also increase with increasing image width. Although this provides some insight on the behavior of S_min_, it is important to note that linear superposition is not valid in general (for example, see Rother et al. [12]). Also, all of the images we show are computed using our model, which can accommodate arbitrary object combinations. In no cases do we assume linear superposition of images due to individual objects.

For a given pair of objects, the rostral object's center coordinates were chosen as the spatial location for which the S_min_ was determined. Therefore, this object was held stationary during a given S_min_ measurement. The caudal object was moved systematically in the caudal direction until two distinct peaks appeared in the electric image (object center-to-skin distance was kept constant). Using this technique, S_min_ measurements were accurate to within 0.5 or 1 mm, representing the chosen sampling (see error bars in Figure 2).

In the last part of the paper, where fish motion is simulated, a scanning speed of 0.1 m/s was chosen, which is in the range of measured weakly electric fish scanning velocities [45,56]. For quantifying the SNR between the two different transdermal potential time series (Figure 5, green and blue curves), i.e., the ones produced by the background alone (Φ*^back^*) and by the background and prey (Φ*^back^* ^+*prey*^), a root-mean-squared difference measure was used (Equation 1):

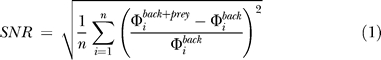
where *n* represents the number of different fish locations that were simulated, i.e., samples of the transdermal potential at a given body location during a 1-s scan (we chose *n* = 21). A large SNR value means that the two time series are very distinct. We have also quantified the discriminability of two objects as they are separated (Equation 2). Here, we assumed that the separate (simulated) electric images generated by each object is a spatial Gaussian function (along one dimension; each of mean μ_i_ and width σ_i_) and have computed the discriminability *d*′ [60,61]:

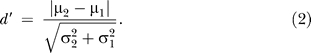



## Abbreviations

ELL: electrosensory lateral line lobe
EO: electric organ
SNR: signal-to-noise ratio
